# Supplementary material for: Particle Shape Influences Settling and Sorting Behavior in Microfluidic Domains
Source: Sci Rep. 2018 Jun 5;8:8583. doi: 10.1038/s41598-018-26786-7 (PMC5988840; doi:10.1038/s41598-018-26786-7)
Supplement: Supplementary file 1 — Supplementary Information [file 41598_2018_26786_MOESM1_ESM.pdf]

Supplementary Information

Particle Shape Influences Settling and Sorting Behavior in Microfluidic Domains

Hakan Başağaoğlu [1,*], Sauro Succi[2], Danielle Wyrick[3], and Justin Blount[4]

[1]Mechanical Engineering Division, Southwest Research Institute, San Antonio, TX 78238 USA
[2]Istituto Applicazioni del Calcolo, via dei taurini 19, 00185, Roma, Italy
[3]Space Science Division, Southwest Research Institute, San Antonio, TX 78238 USA
[4]Defense Intelligence Solutions Division, Southwest Research Institute, San Antonio, TX 78238 USA
[*]Corresponding author: hbasagaoglu@swri.org

ABSTRACT

Geometric descriptions of the boomerang-, triangular-, hexagonal-, rectangular-, and circular-shaped particles are provided in Supplementary Information-1. Numerically computed shape-dependent settling (terminal) velocities of particles are discussed in Supplementary Information-2. The effect of grid resolution on the simulation results are presented in Supplementary Information-3. Transient flow trajectories of a circular particle in a Poiseuille flow at different Reynolds numbers, Re, are shown in Supplementary Information-4. The steric interparticle and particle-wall interactions are described in Supplementary Information-5. Numerically simulated flow trajectories of four representative particles in a microfluidic device geometry, shown in Fig.9, are provided in Supplementary Information-6.

Supplementary Information-1: Geometric Description of DSP

Boomerang-shaped Particle

The boomerang-shaped particle geometry is described by four vertices $v_{b1} \cdots v_{b4}$, and three angles, where α, ϕ, and ζ (Fig. 1c). α is the initial tilt angle of the particle in the counter-clockwise direction, and ϕ and ζ specify the spatial variations of the width of the wings. The particle geometry is generated by carving out a small isosceles triangle, BDC, from a large isosceles triangle, BAC. The locations of vertices of the boomerang-shaped particle are computed by

$$\begin{bmatrix} x_{v_{B1}} \\ y_{v_{B1}} \\ x_{v_{B2}} \\ y_{v_{B2}} \\ x_{v_{B3}} \\ y_{v_{B3}} \\ x_{v_{B4}} \\ y_{v_{B4}} \end{bmatrix} = \begin{bmatrix} x_c \\ y_c \\ x_c \\ y_c \\ x_c \\ y_c \\ x_c \\ y_c \end{bmatrix} - \frac{B}{4} \begin{bmatrix} 2cos(\alpha) + \eta_c + \varphi_c \\ 2sin(\alpha) + \eta_s + \varphi_s \\ 2cos(\alpha) + \eta_c - 3\varphi_c \\ 2sin(\alpha) + \eta_s - 3\varphi_s \\ \eta_c + \varphi_c - 6cos(\alpha) \\ \eta_s + \varphi_s - 6sin(\alpha) \\ 2cos(\alpha) + \varphi_c - 3\eta_c \\ 2sin(\alpha) + \varphi_s - 3\eta_s \end{bmatrix}, \tag{1}$$

which $\eta_c = cos(\alpha + \zeta)/cos(\zeta)$, $\varphi_c = cos(\alpha + \phi)/cos(\phi)$, $\eta_s = sin(\alpha + \zeta)/cos(\zeta)$, and $\varphi_s = sin(\alpha + \phi)/cos(\phi)$. The mass of the boomerang-shaped particle per unit particle thickness is given by $m_p = A_B \rho_p$, in which the surface area of the boomerang-shaped geometry is $A_B = B^2 \left[tan(\zeta) - tan(\phi)\right]$ and B is half of the base length of the triangle BDC. The particle's moment of inertia is computed via $I_p = \frac{m_p}{72}\left[4\left(H^2 + Hh + h^2\right) + 3B^2\right] - \kappa^2 A_{BDC}$, where H and h are the heights of the triangle BAC and BDC, in which $H = Btan(\zeta)$ and $h = Btan(\phi)$, κ is the distance between the center of mass of the triangle BAC and triangle BDC, and A_{BDC} is the area of the triangle BDC.

Equilateral Triangular-shaped Particle

The equilateral triangular particle geometry is represented by the side length of a, three vertices, $v_{T1} - v_{T3}$, and an initial tilt angle, α, in the counter-clockwise direction (Fig. 1d). The coordinates of the vertices of the triangular particle are given by

$$\begin{bmatrix} x_{v_{T1}} \\ y_{v_{T1}} \\ x_{v_{T2}} \\ y_{v_{T2}} \\ x_{v_{T3}} \\ y_{v_{T3}} \end{bmatrix} = \begin{bmatrix} x_c \\ y_c \\ x_c \\ y_c \\ x_c \\ y_c \end{bmatrix} + \frac{2h}{3} \begin{bmatrix} cos\left(\pi/2 + \alpha\right) \\ sin\left(\pi/2 + \alpha\right) \\ -sin\left(\pi/3 - \alpha\right) \\ -cos\left(\pi/3 - \alpha\right) \\ sin\left(\pi/3 + \alpha\right) \\ cos\left(\pi/3 + \alpha\right) \end{bmatrix}, \tag{2}$$

in which h is the height of a triangle, $h = \frac{\sqrt{3}}{2}a$. The particle mass per unit thickness is given by $m_p = A_T \rho_p$, in which the surface area of the equilateral triangle is $A_T = \frac{\sqrt{3}}{4}a^2$. The moment of inertia of a triangular particle is computed from $I_T = \frac{m_p}{72}\left(3a^2 + 4h^2\right)$.

Hexagonal-shaped Particle

The hexagonal particle geometry is described by an uniform side length of L and initial tilt angle, α (Fig. 1e). The locations of vertices of the hexagonal particle, $v_{H1} \cdots v_{H6}$, are computed by

$$\begin{bmatrix} x_{v_{Hi}} \\ y_{v_{Hi}} \end{bmatrix} = \begin{bmatrix} x_c \\ y_c \end{bmatrix} + L \begin{bmatrix} cos\left(\alpha + (i-1)\pi/3\right) \\ sin\left(\alpha + (i-1)\pi/3\right) \end{bmatrix}, \tag{3}$$

in which $i \varepsilon [1, 6]$. The mass of a hexagonal-shaped particle per unit particle thickness is $m_p = A_H \rho_p$, in which the surface area of the particle is $A_H = \frac{3\sqrt{2}}{3}L^2$. The moment of inertia of a hexagonal particle is $I_p = \frac{m_p L^2}{24}\left[1 + 3cot^2\left(\frac{\pi}{6}\right)\right]$.

Rectangular-shaped Particle

The rectangular-shaped particle geometry is represented by two side lengths of l and w, four vertices, $v_{R1} - v_{R4}$, and an initial tilt angle, α, in the counter-clockwise direction (Fig. 1f). The coordinates of the vertices are

$$\begin{bmatrix} x_{v_{R1}} \\ y_{v_{R1}} \\ x_{v_{R2}} \\ y_{v_{R2}} \\ x_{v_{R3}} \\ y_{v_{R3}} \\ x_{v_{R4}} \\ y_{v_{R4}} \end{bmatrix} = \begin{bmatrix} x_c \\ y_c \\ x_c \\ y_c \\ x_c \\ y_c \\ x_c \\ y_c \end{bmatrix} + \frac{\sqrt{l^2+w^2}}{2} \begin{bmatrix} cos\left(\alpha+\theta\right) \\ sin\left(\alpha+\theta\right) \\ -cos\left(\alpha-\theta\right) \\ sin\left(\alpha-\theta\right) \\ -cos\left(\alpha+\theta\right) \\ -sin\left(\alpha+\theta\right) \\ cos\left(\alpha-\theta\right) \\ -sin\left(\alpha-\theta\right) \end{bmatrix}. \tag{4}$$

The particle mass per unit thickness was given by $m_p = A_R \rho_p$, in which the surface area of the rectangular geometry is $A_R = lw$. The moment of inertia of a rectangular particle is computed from $I_R = \frac{m_p}{12}\left(l^2+h^2\right)$.

Circular-shaped Particle

The circular particle geometry is constructed by equally-spaced boundary nodes, N_{bnd}, along its curved surface and the particle radius, R (Supplementary Fig. 1). The coordinates of the boundary nodes (denoted by small circles in Supplementary Fig. 1) are computed by

$$\begin{bmatrix} x_i \\ y_i \end{bmatrix} = \begin{bmatrix} x_c \\ y_c \end{bmatrix} + R \begin{bmatrix} cos\left(2\pi\left(i-1\right)/\left(N_{bnd}-1\right)\right) \\ sin\left(2\pi\left(i-1\right)/\left(N_{bnd}-1\right)\right) \end{bmatrix}. \tag{5}$$

The mass of the circular particle per unit particle thickness is $m_p = A_C \rho_p$, in which the surface area of the circular particle is $A_C = \pi R^2$. When the circular particle is treated as a thin solid disk, its moment of inertia is computed via $I_C = \frac{1}{2} m_p R^2$.

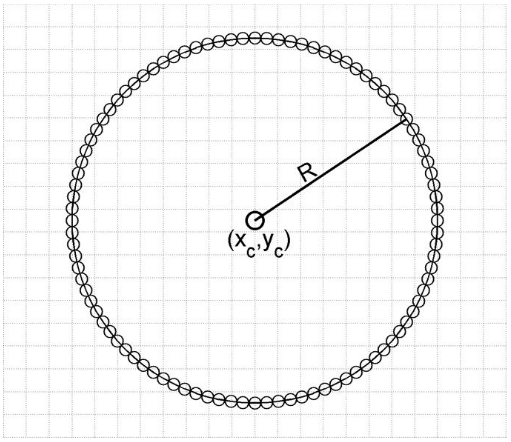

Supplementary Figure 1. A schematic representation of a circular particle geometry in the LBM.

Supplementary Information-2: Settling (Terminal) Velocities of DSP

The settling velocity of a particle approaches a constant (equilibrium) value as the buoyant force is balanced by the viscous drag force[1]. The DSP-LBM was used to calculate the effect of particle shapes on the settling (terminal) velocities, U_s, of DSP, as a function of particle density and the blockage ratio, W/R_e, in which W is the channel width and R_e is the effective particle radius as described in the main text. From a series of experiments conducted with glass spheres ranging in size from 0.1 μm to 6 mm in diameter, an empirical relation (Eq. 6) was constructed by Gibbs *et al.*[2] to determine the settling velocity of spherical particles in an initially quiescent water in a bounded domain,

$$U_s = \frac{-3\rho\nu + \sqrt{9(\rho\nu)^2 + |\mathbf{g}|R_p^2\rho(\rho_p-\rho)(0.015476+0.19841R_p)}}{\rho(0.011607+1.14881R_p)}. \tag{6}$$

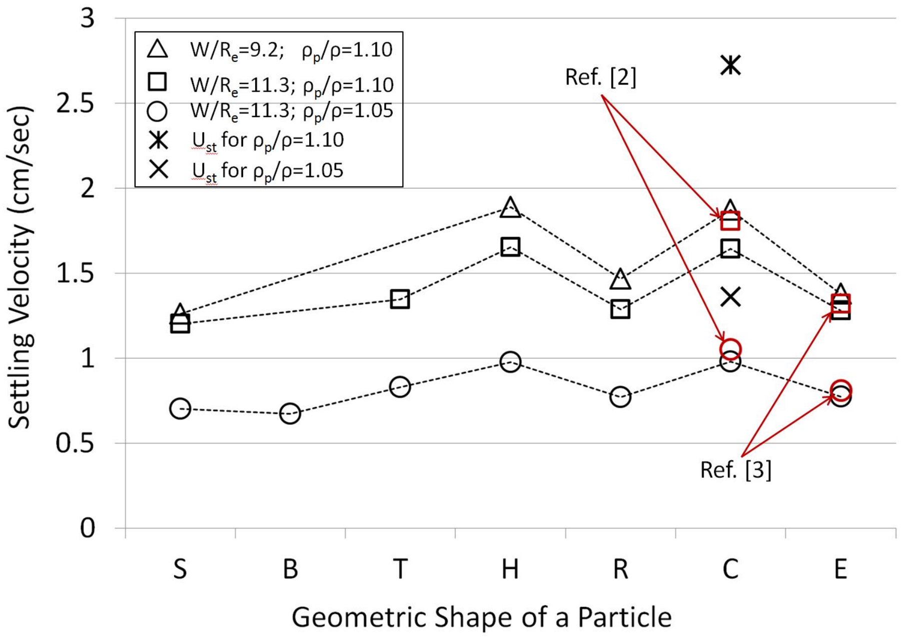

Supplementary Figure 2. DSP-LBM simulations of settling (terminal) velocities, U_s, of DSP with different densities and blockage ratios. The letters in the horizontal axis corresponds to the first letter of particle geometries. Red symbols correspond to the literature data.

U_s computed by Eq. 6 for a spherical particle with $\rho_p/\rho = 1.05$ and 1.1 is in a good agreement with the settling velocities of a circular particle with ρ_p/ρ =1.05 and $\rho_p/\rho = 1.10$ computed by the DSP-LBM in Supplementary Fig. 2. Moreover, the settling velocities of an elliptical particle for $\rho_p/\rho = 1.05$ and 1.1 computed by the DSP-LBM are in good agreement with particle settling velocities reported by Xia *et al.*[3].

Supplementary Fig. 2 also reports U_s for the circular particle computed by the Stokes equation, given by $U_{st} = (\rho_p-\rho)(2R_p)^2/18(\rho\nu)$. U_{st} overestimated U_s computed by the DSP-LBM and Eq. 6 by 25% for ρ_p/ρ =1.05 and 47% for ρ/ρ_p =1.10, and hence, the settling of the particle with ρ_p/ρ =1.05 and ρ_p/ρ =1.10 cannot be correctly expressed by Stokes equation. The results shown in Fig. 2 also revealed that the settling velocities of the circular, rectangular, and hexagonal particles were more sensitive to the blockage ratio than those of star and elliptical particles.

Supplementary Information-3: Grid Resolution

To demonstrate the insensitivity of the settling velocities and trajectories of DSP to grid resolution, grid resolution was doubled (2x-Resolution) such that the surface area of each particle was represented by 530 lattice cells, as compared to 265 lattice cells for the base case (x-Resolution) in DSP-LBM simulations. The resultant discrepancies in the settling velocities of DSP were within 2% (Supplementary Table 1).

Supplementary Table 1. Settling velocities of DSP (in cm/s) for $\rho_p/\rho = 1.05$ at $x/W = 20$, as a function of a grid resolution.

	Star	Boomerang	Triangular	Hexagonal	Rectangular	Circular	Elliptical
x-Resolution	0.00900	0.00865	0.01064	0.01250	0.00987	0.01254	0.00990
2x-Resolution	0.00899	0.00856	0.01051	0.01243	0.01007	0.01268	0.00998
% discrepancy	0.15	1.07	1.26	0.58	1.94	1.10	0.82

The trajectories of the boomerang, triangular, elliptical, and rectangular particles with $\rho_p/\rho = 1.05$ for x- and 2x-Resolutions are shown in Supplementary Fig. 3. For $\rho_p/\rho = 1.05$, star-shaped and hexagonal shaped particles settled near the centerline, but their settling trajectories exhibited relatively larger lateral displacements when $\rho_p/\rho = 1.10$. Therefore, the sensitivity of their trajectories to grid resolution is shown for $\rho_p/\rho = 1.10$ in Supplementary Fig. 4.

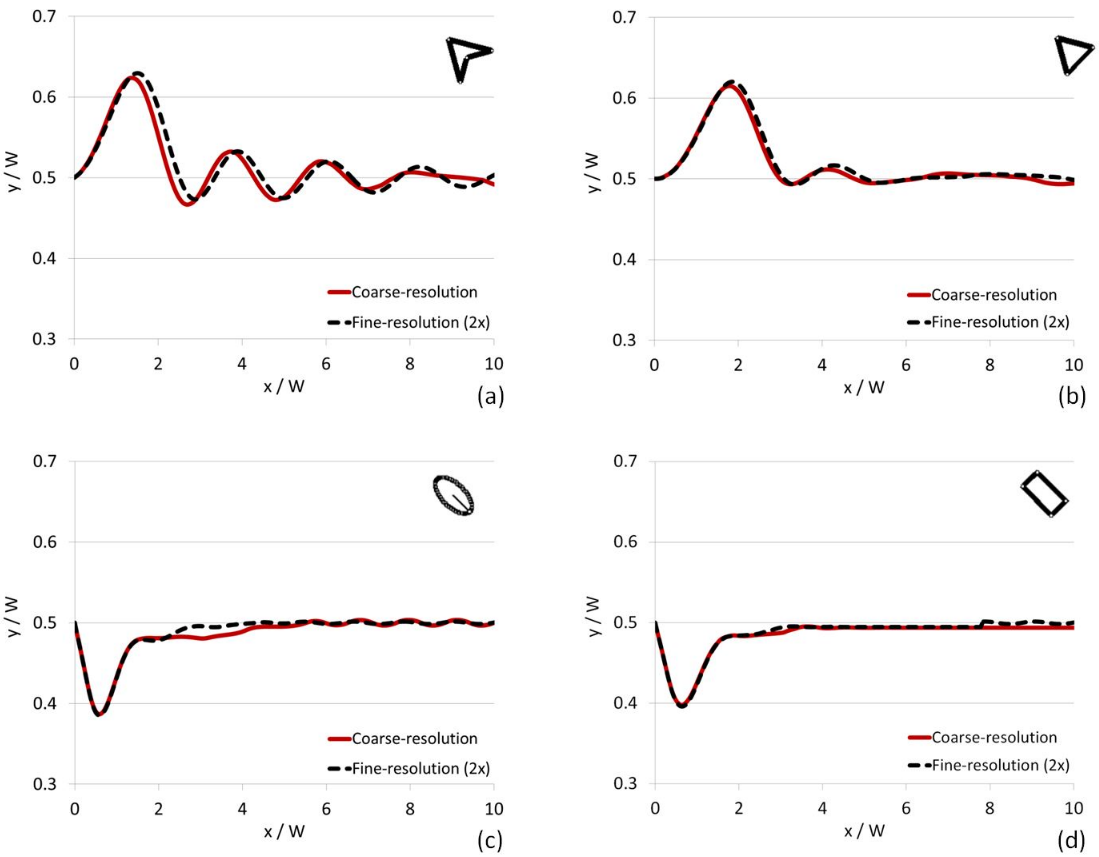

Supplementary Figure 3. The effect of grid resolution on the trajectories of a (a) boomerang, (b) triangular, (c) elliptical and (d) rectangular particle for $\rho_p/\rho = 1.05$.

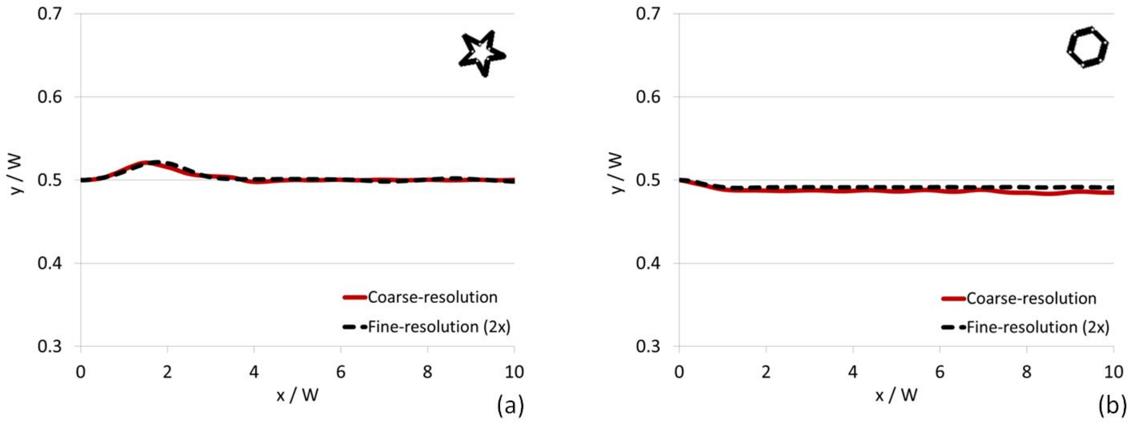

Supplementary Figure 4. The effect of grid resolution on the trajectories of a (a) star and (b) hexagonal particle for $\rho_p/\rho = 1.10$.

In brief, the general trend of settling trajectories of DSP at the relatively coarser and two-times finer resolutions were similar, as shown in Supplementary Figs. 3 and 4. Moreover, the settling velocities of DSP at the finer and coarser resolutions differed only by $\leq 2\%$ (Supplementary Table 1). Therefore, the coarser grid resolution was deemed sufficient for numerical simulations in this paper.

Supplementary Information-4: Flow Trajectories of a Circular-Cylindrical Particle in a Poiseuille Flow

Flow trajectories of a neutrally-buoyant circular particle in a Poiseuille flow at different *Re* are shown in Supplementary Fig. 5. In these simulations, $2R_p = 0.08$ cm, $\nu = 0.01$ cm^2/s, and $W/R_p = 11.3$.

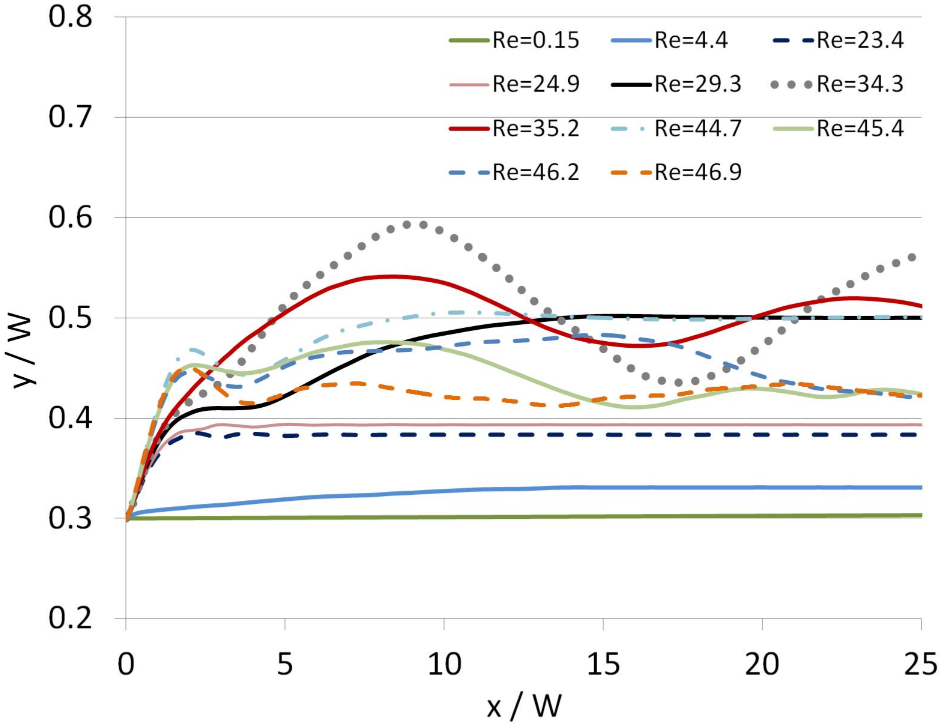

Supplementary Figure 5. Flow trajectories of a neutrally-buoyant circular particle in Poiseuille flow at different *Re*.

Supplementary Information-5: Particle-Particle and Particle-Wall Steric Interaction Forces

Steric interaction forces, $\mathbf{F}_{\mathbf{r}_i}$, between the particles and between the particles and stationary solid zones, including channel walls and inline obstacles, are expressed in terms of two-body Lennard-Jones potentials[4] such that $\mathbf{F}_{\mathbf{r}_i} = -\psi\left(\frac{|\mathbf{r}_i|}{|\mathbf{r_{it}}|}\right)^{-13}\mathbf{n}$, where $|\,\mathbf{r}_i\,|$ is the distance between a particle surface node and the neighboring particle surface node ($\mathbf{r}_i = \mathbf{r}_{pp'}$) or between a particle surface node and the stationary solid node located on channel walls or inline obstacles ($\mathbf{r}_i = \mathbf{r}_{pw}$); p is the particle index; $|\,\mathbf{r}_{it}\,|$ is the repulsive threshold distance; $\mathbf{n}$ is the unit vector along $\mathbf{r}_i$; and ψ is the stiffness parameter used to adjust the repulsive strength between the particles and between the particles and stationary solid zones. The total particle-fluid hydrodynamic forces are computed by

$$\mathbf{F}_T = \sum_{\mathbf{r}_b}\mathbf{F}_{\mathbf{r}_b} + \sum_{\mathbf{r}_b^{c,u}}\mathbf{F}_{\mathbf{r}_b^{c,u}} + \sum_{|\mathbf{r}_{pw}|\leq|\mathbf{r}_{it}|}\mathbf{F}_{\mathbf{r}_{pw}} + \sum_{|\mathbf{r}_{pp'}|\leq|\mathbf{r}_{it}|}\mathbf{F}_{\mathbf{r}_{pp'}}, \quad (7)$$

in which the first term on the right-hand-side of Eq. 7 is used to calculate particle-fluid hydrodynamic forces at the surface (boundary) nodes located at $\mathbf{r}_b$, the second term is used to calculate forces associated with the uncovered or covered lattice nodes at $\mathbf{r}_b^{c,u}$ due to particle motion, the third term is used to calculate interparticle steric interaction forces, and the fourth term is used to calculate the steric interaction forces between the particles and stationary solid zones. Please see Ref.[4] for a more detailed explanation of these terms.

In this formulation, the total steric interaction forces on a particle exerted by neighboring particles scale with the number of surface nodes of neighboring particles enclosed by an envelope $|\,\mathbf{r}_{it}\,|$ around the particle of interest. The same scaling is also applicable for the steric interactions between the particles and stationary solid zones. In our simulations, $|\,\mathbf{r}_{it}\,| = 2.5$ lattice unit (l.u.) away from particle surface and $\psi = 1$. Interparticle steric interaction forces are non-zero only when surface boundary nodes of neighboring particles are within 2.5 l.u. of the particle of interest to avoid physically unrealistic overlaps.

Let the total particle-fluid hydrodynamic force, not excluding the interparticle and particle-wall steric interaction forces, be $\mathbf{F}_H = \sum_{\mathbf{r}_b}\mathbf{F}_{\mathbf{r}_b} + \sum_{\mathbf{r}_b^{c,u}}\mathbf{F}_{\mathbf{r}_b^{c,u}}$ and interparticle steric interaction forces be $\mathbf{F}_{PP} = \sum_{|\mathbf{r}_{pp'}|\leq|\mathbf{r}_{it}|}\mathbf{F}_{\mathbf{r}_{pp'}}$. In our simulations, $|\,\mathbf{F}_H\,|$ is typically on the order of $|\,\mathbf{F}_{pp}\,|$, if the surface (boundary) nodes of neighboring particles are separated from boundary nodes of the particle of interest by a distance $|\,\mathbf{d}\,|$ such that 1 l.u. $\leq |\,\mathbf{d}\,| \leq$ 2.5 l.u. However, if $|\,\mathbf{d}\,| < 1.0$, $\mathbf{F}_{\mathbf{r}_{pp}}$ results in an instantaneous, short-lived relatively large steric pulse to keep the separation distance larger than 1 l.u. (Fig. 6). The movie file showing the settling of a mixture of DSP in Figs. 8a-b, using the interparticle and particle-wall steric interactions in Eq. 7, is provided as a Supplementary Movie file.

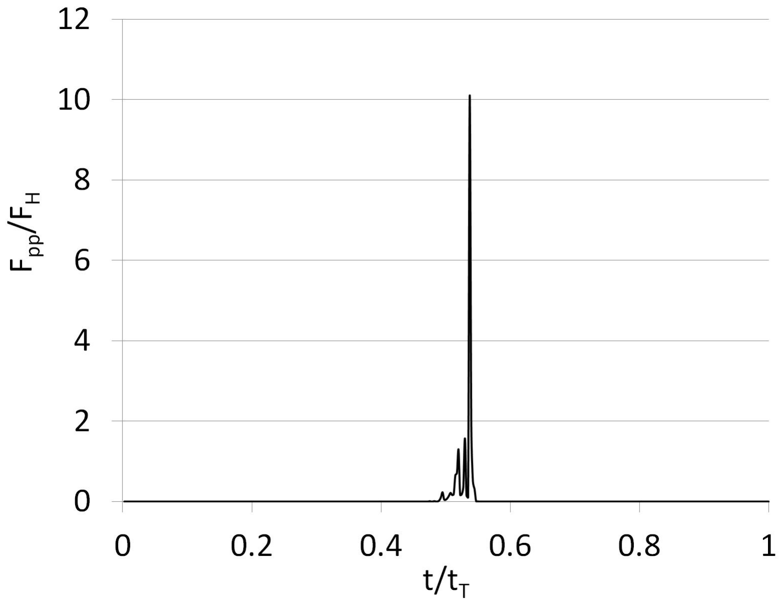

Supplementary Figure 6. The ratio of the interparticle steric interaction forces to the total particle-fluid hydrodynamics forces, excluding steric interaction forces, acting on the settling elliptical particle. t_T is the total simulation time.

Supplementary Information-6: Particles Trajectories in a Microfluidic Device

Particles of different shapes and sizes were released into water after the steady flow field was established in a microfluidic device geometry in Fig. 9. Transient flow trajectories of four of these particles are shown in Supplementary Fig. 7, which reveals that neither large particles nor small particles were permanently trapped in steady vortex structures established in the fluid prior to releases of the particles.

The particle L1 (L represents the large particles) was trapped temporarily in the upper and lower halves during its first trip, but eventually escaped the entrapments and left the flow domain. In its second trip, it was trapped in the lower half. Unlike the particle L1, the particle L10, completed the first three trips without being trapped but got trapped in the lower half in its fourth trip. The particle S12 (S represents small particles) completed its first trip with relatively short-lived entrapments, avoided entrapments in its second and third trips, but displayed a prolonged entrapment in its fourth trip. The particle S22, on the other hand, exhibited entrapments in its first and third trips, but flew smoothly in its second trip without any entrapments.

Numerical simulations revealed similar flow behaviors for all 40 particles. None of these particles were permanently entrapped in transient vortices or in steady vortex regions. The flow field was inherently transient after releases of particles into an initially steady flow field in Fig. 9. The location, size, and number of vortex structures continuously altered as the mobile particles continuously exchanged momentum with the fluid. Hence, the steady vortex structures in Fig. 9 are not responsible for particle entrapments in this simulation.

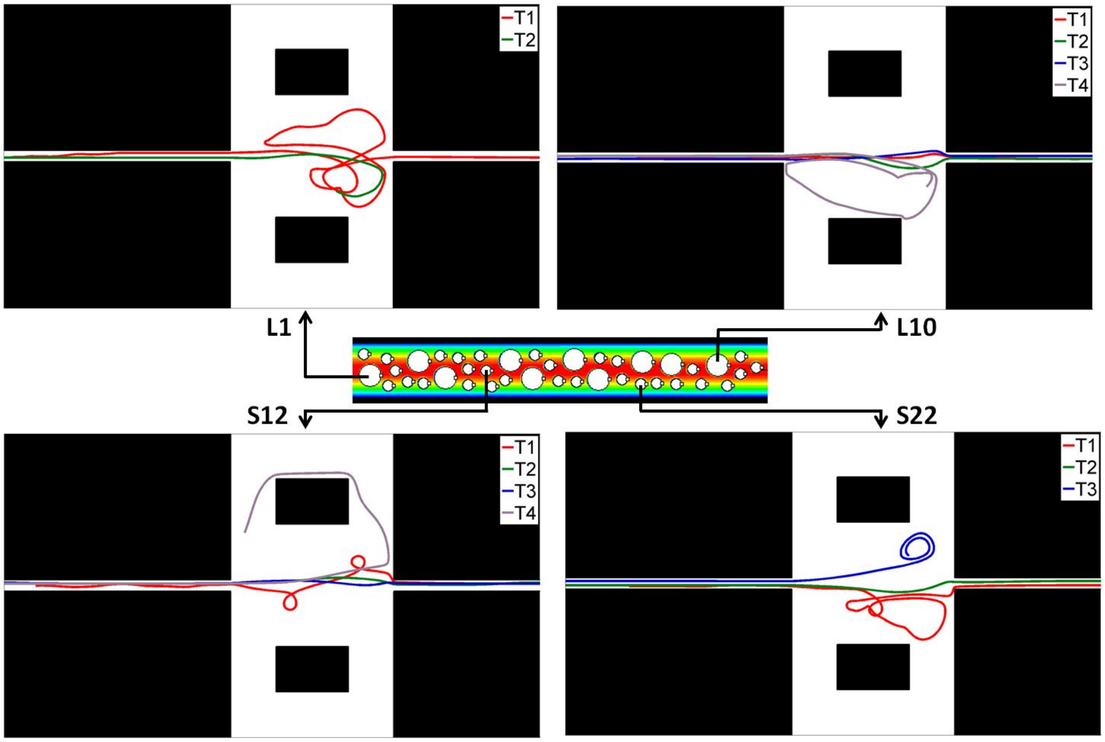

Supplementary Figure 7. Flow trajectories of four particles in a microfluidic chamber after the steady flow field was established. T(i) refers to the (i)th trip of the particle in the microfluidic chamber. L denotes large particles and S denotes small particles. Particles leaving the domain from the exit-end were allowed to re-enter the flow domain from the inlet.

References

1. Mougin, G. & Magnaudet, J. Path instability of a rising bubble. *Phys Rev Lett* **88**, 014502 (2002).
2. R.J. Gibbs, M.D. Matthews & D.A. Link. The relationship between shpere size and settling velocity. *J Sedimentary Petrol* **41**, 7–18 (1971).
3. X.Zhenhua *et al.* Flow patterns in the sedimentation of an elliptical particle. *J Fluid Mech* **625**, 249–272 (2009).
4. Başağaoğlu, H. & Succi, S. Lattice-Boltzmann simulations of repulsive particle-particle and particle-wall interactions: Coughing and choking. *J Chem Phys* **132**, 134111 (2010).
